# Supplementary material for: Response to anxiety treatment before, during, and after the COVID-19 pandemic
Source: PLoS One. 2024 Mar 13;19(3):e0296949. doi: 10.1371/journal.pone.0296949 (PMC10936764; doi:10.1371/journal.pone.0296949)
Supplement: Attachment — Submitted filename: 20230913_Response.docx [file pone.0296949.s001.docx]

September 13, 2023

Dear Editors,

Thank you for your stewardship of the review process of our manuscript *Response to Anxiety Treatment Before, During, and After the COVID-19 Pandemic* for publication as a Research Article in *PlosOne.* We are grateful to reviewers for their helpful comments, which we believe have improved the paper.

Below we detail responses to reviewers, and changes made to our manuscript. Thank you for considering it for publication.

David H. Rosmarin

Reviewer #1:

Please adding more relevant key words

- *Thank you, we have added several key words.*

Please consistently use same format in describing the dates such as July 1st 2020 throughout the article.

- *We are grateful for this point, and have made the dates consistent throughout the manuscript.*

The result and discussion only highlighted about the efficacy of CBT for anxiety but in the methodology indicated that the respondents also received DBT.

- *CBT is a large conglomerate of therapy techniques that includes DBT. However, we appreciate Reviewer #1’s point and have mentioned these approaches separately in the paper.*

Please mention how many sessions your respondents received and is there any standardized protocol to for your intervention. Please discuss the medium that you used in conducting your intervention.

- *We have added the number of weekly sessions completed by each study group to Table 1. We have also clarified in the introduction and methods that patients presented to a naturalistic outpatient setting. Finally, we have added to the discussion that this feature of our study adds to the ecological validity of the findings.*

Please explain how you compare the unequal group size and controlled the possible bias.

- *Multilevel modeling is robust to unequal group sizes. We have added a brief comment to this effect at the start of the Statistical Analyses section.*

Reviewer #2: The role of CBT in anxiety treatment is an important area of research in mental health studies. The present article will add to the research in this area.

- *Thank you for these kind comments.*

There are a few clarifications needed from the authors:

1. Was there uniformity of treatment in all groups? Did the "during pandemic" and "post

pandemic " group receive any additional counselling, specific to the pandemic? Was their

counselling more frequent?

- *Our results suggest that groups received a similar number of sessions, relative to their aggregate time in therapy.*

2. The data in this study is of anxiety patients who adhered to treatment and completed it. There is no mention of any data of patients who had dropped out of treatment during the specified period. It would be pertinent to know the proportion of anxiety patients who started the treatment but did not complete it.

- *As above comments to Reviewer #1, we have added the number of treatment sessions completed by each group. Note that the naturalistic nature of our study precludes assessment of “drop-outs” since there was no fixed start or end point for treatment.*
